# Optimization of Potential Nanoemulgels for Boosting Transdermal Glimepiride Delivery and Upgrading Its Anti-Diabetic Activity

**DOI:** 10.3390/gels9060494

**Published:** 2023-06-18

**Authors:** Marwa H. Abdallah, Amr S. Abu Lila, Hanan M. El-Nahas, Tarek M. Ibrahim

**Affiliations:** 1 Department of Pharmaceutics, College of Pharmacy, University of Ha’il, Hail 81442, Saudi Arabia; 2 Department of Pharmaceutics, Faculty of Pharmacy, Zagazig University, Zagazig 44519, Egypt; hananelnahas@gmail.com (H.M.E.-N.); tarekmetwally333@gmail.com (T.M.I.)

**Keywords:** diabetes, glimepiride, nanoemulsion, pharmacokinetics, transdermal delivery

## Abstract

Transdermal drug delivery has been widely adopted as a plausible alternative to the oral route of administration, especially for drugs with poor systemic bioavailability. The objective of this study was to design and validate a nanoemulsion (NE) system for transdermal administration of the oral hypoglycemic drug glimepiride (GM). The NEs were prepared using peppermint/bergamot oils as the oil phase and tween 80/transcutol P as the surfactant/co-surfactant mixture (S_mix_). The formulations were characterized using various parameters such as globule size, zeta potential, surface morphology, in vitro drug release, drug-excipient compatibility studies, and thermodynamic stability. The optimized NE formulation was then incorporated into different gel bases and examined for gel strength, pH, viscosity, and spreadability. The selected drug-loaded nanoemulgel formulation was then screened for ex vivo permeation, skin irritation, and in vivo pharmacokinetics. Characterization studies revealed the spherical shape of NE droplets with an average size of ~80 nm and a zeta potential of −11.8 mV, which indicated good electrokinetic stability of NE. In vitro release studies revealed enhanced drug release from the NE formulation compared to the plain drug. GM-loaded nanoemulgel showed a 7-fold increment in drug transdermal flux compared to plain drug gel. In addition, the GM-loaded nanoemulgel formulation did not elicit any signs of inflammation and/or irritation on the applied skin, suggesting its safety. Most importantly, the in vivo pharmacokinetic study emphasized the potential of nanoemulgel formulation to potentiate the systemic bioavailability of GM, as manifested by a 10-fold rise in the relative bioavailability compared to control gel. Collectively, transdermal NE-based GM gel might represent a promising alternative to oral therapy in the management of diabetes.

## 1. Introduction

Diabetes type 2 is a chronic metabolic condition identified by elevated blood glucose levels. It is a lifelong illness that requires close monitoring and changes in the patient’s lifestyle [1]. The prevalence of diabetes mellitus has risen globally and in particular in Saudi Arabia. According to the International Diabetes Federation, Saudi Arabia is ranked as the seventh-highest country for new cases of diabetes per year, with a prevalence approaching 20% among adults [2]. This worrisome surge in diabetes statistics is due to a global shift toward urban lifestyles that are characterized by an unbalanced diet and decreased physical exercise. The U.S. Food and Drug Administration indicates that proper diet, physical activity, and medications are appropriate medical interventions for the treatment of diabetic patients [3].

Glimepiride (GM) is an oral hypoglycemic agent belonging to the class of drugs known as sulfonylureas. It works by boosting insulin production from pancreatic beta cells [4] and improving intracellular insulin sensitivity [5]. GM is commonly prescribed to treat high blood glucose levels caused by type 2 diabetes. It can be used alone or in conjunction with insulin or other oral hypoglycemic agents such as metformin [6,7]. Nevertheless, its oral administration is associated with limited effectiveness, presumably due to its low aqueous solubility and sluggish dissolution rate, resulting in limited oral bioavailability [8,9]. To address these drawbacks, several approaches have been adopted to enhance GM solubility, including solid dispersions [10], inclusion complexation [11], cosolvency [12], nanocrystals [13], and spray congealing [14]. However, these approaches have had limited success [15].

Transdermal drug delivery constitutes an appealing alternative to conventional drug delivery systems [16,17]. It provides the advantage of being a non-invasive and convenient route of administration. In addition, the skin can provide a “reservoir” that sustains the delivery of drugs for prolonged periods [18,19]. Furthermore, transdermal drug delivery can offer additional advantages such as minimizing fluctuations in plasma drug levels, avoiding first-pass metabolism, and improving patient compliance [19,20]. Nevertheless, the clinical use of transdermal delivery is adversely restrained by the fact that only a few drugs have the ability to penetrate the skin barrier layer to exert a systemic effect [21].

The application of nanocarriers has emerged as a feasible means for overcoming the constraints of transdermal treatment [22]. Nanocarriers have been viewed as viable delivery vehicles for transdermal administration because of their tiny particle size, improved drug retention, and targeting capabilities. As a result, several techniques have been developed to improve transdermal administration of bioactive drugs employing nanoparticulate drug delivery systems such as lipid-based nanovesicles [20], polymeric nanoparticles [23], dendrimers [24], and micro-/nano-emulsions [25,26]. Among them, nanoemulsions (NEs) are regarded as heterogeneous colloidal mixtures of oily and aqueous phases associated with a surfactant adsorbed at the interface between both phases. Such surfactants can help decrease the surface tension and enhance the system’s stability [27]. As efficacious delivery systems, NEs can exhibit several merits, including great solubilization capacity, high drug loading, controlled drug release profiles, and the potential delivery of lipophilic drugs. Nevertheless, owing to their low viscosity and spreadability and inadequate skin retention, NEs are commonly incorporated into gelling agents to be modified into nanoemulgels [28].

The nanoemulgel is regarded as a potential transdermal drug delivery system due to its dual nature—the existence of a nanosized emulsion and a gel base, both mixed in a single formulation [29]. In contrast to hydrogels, which have difficulty transporting hydrophobic medications, nanoemulgels can carry both hydrophilic and lipophilic drugs efficiently. Furthermore, as compared to emulgels, nanosized emulsion droplets provide nanoemulgels with a large surface area, allowing for efficient penetration through skin pores and effective transport of the loaded medication into systemic circulation. Additionally, by adjusting the emulsion bases in nanoemulgels, the bioadhesive characteristics and drug-release pattern may be fine-tuned for a sustained therapeutic effect [30]. Most importantly, as compared to other nanocarriers, nanoemulgel has a higher drug loading capacity, greater penetration/diffusion, and less skin irritation [28]. Accordingly, many anti-diabetic drugs were successfully loaded within the nanoemulgel systems, such as insulin [31], glibenclamide [32], repaglinide [33], and glimepiride [34].

Therefore, in this study, we aimed at enhancing the ability of GM to permeate through the skin layer to ensure higher drug therapeutic activity. For this purpose, the solubility of GM in different NE components was measured, followed by the validation and screening of various surfactants and co-surfactants. Several GM-loaded NE-based systems were developed to study the effect of changing the concentrations of NE components on the characteristics of NE systems using the CCD. Afterwards, the optimized GM-loaded NE formulation was incorporated into different hydrogel bases, and physicochemical, ex-vivo skin permeation, and histopathological studies were carried out. Finally, the in vivo pharmacokinetic studies of the optimized GM-loaded nanoemulgel formulation after transdermal application into rats were investigated to examine its feasibility for boosting the transdermal delivery of GM and upgrading its anti-diabetic activity in rats.

## 2. Results and Discussion

### 2.1. Screening of NE Components

The selection of an oily phase is crucial since it indirectly affects drug loading and keeps it dissolved in the NE system [35]. Accordingly, the solubility of GM in different oils, including peppermint, bergamot, mandarin, tea tree, eucalyptus, fennel, lavender, rosemary, orange, and lemon oils, was assessed. As depicted in Table S1, the maximum solubility of the drug was observed in both peppermint oil (3.67 ± 0.13 mg/g) and bergamot oil (2.75 ± 0.91 mg/g). While the minimum solubility of GM was observed with orange and lemon oils: 0.5 ± 0.24 mg/g and 0.43 ± 0.09 mg/g, respectively. Accordingly, peppermint and bergamot oils, individually or in a mixture, were chosen as the oil phase.

Surfactants and co-surfactants help to reduce interfacial tension between the oily phase and the aqueous phase of NE by being adsorbed at the o/w interface and acting as a mechanical barrier to coalescence. Generally, during the formulation of NEs, non-ionic surfactants are preferred over their ionic counterparts since ionic surfactants usually suffer from bioactivity and toxicity issues, while non-ionic surfactants are viewed as safe because of their hydrophilic nature [34,36]. Consequently, the solubilization power of non-ionic surfactants such as spans, tweens, labrafil isostearic, and labrafil M 1944 CS was examined. As summarized in Table S1, the solubilization power of the tested surfactants was in the following order: tween 80 > tween 20 > span 20 > labrafil isostearic > span 80 > labrafil M 1944 CS, with a maximum solubilization power observed with tween 80 (2.94 ± 0.09 mg/g). The higher solubility of GM in tween 80 could be related to its greater power to reduce droplet size than the other surfactants owing to its low molecular weight [37]. Accordingly, tween 80 was chosen for further studies.

To generate NE systems with low surfactant concentrations, co-surfactants are added. The co-surfactant is commonly used to reduce bending stress by rendering the interfacial membrane flexible so that it can be deformed easily to efficiently cover each globule [38]. In this study, various co-surfactants, such as transcutol P, PEG 400, PG, ethanol, lauroglycol FCC, and glycerin, were examined for their solubilization power. Among the examined co-surfactants, both transcutol P and PEG 400 were selected for the formulation of NE since they showed the maximum solubilization powers of 2.54 ± 0.20 mg/g and 1.15 ± 0.14 mg/g, respectively. Collectively, based on the solubility profile, peppermint and bergamot oils, tween 80, and transcutol P or PEG 400 were selected as the oil phase, surfactant, and co-surfactant, respectively.

### 2.2. Validation of Surfactant and Co-Surfactant

The assessment of drug solubility in various surfactants and co-surfactants could not be utilized only to identify NE components. Instead, the miscibility of various surfactants and co-surfactants in the oil phase plays a crucial role in the efficient formulation of the NE system. Consequently, the miscibility of several surfactants in peppermint oil or bergamot oil was investigated via sequential additions of a definite volume (5 μL) of either peppermint oil or bergamot oil to an aqueous surfactant solution (2.5 mL of 15% *w*/*w*) until the clear solution became cloudy [39]. As depicted in Table S2, tween 80 demonstrated the greatest miscibility with higher additions of either bergamot oil (275 μL; 55 additions of 5 μL of oil) or peppermint oil (35 μL; 7 additions of 5 μL of oil) without turbidity or phase separation. On the other hand, tween 20 demonstrated lower miscibility in both tested oils with only 6 or 50 additions of 5 μL of either peppermint oil or bergamot oil, respectively. It is well recognized that the difference between the HLB of surfactant and oil dictates the solubility of oil. The higher the difference between the HLB of surfactant and oil, the lower the solubility of surfactant in oil. This might explain the preferential miscibility of tween 80 (HLB = 15) in both bergamot oil (HLB = 12.9) and peppermint oil (HLB = 12.3) in comparison to tween 20, which has a higher HLB value of 16.7 [40].

**Figure 1:**
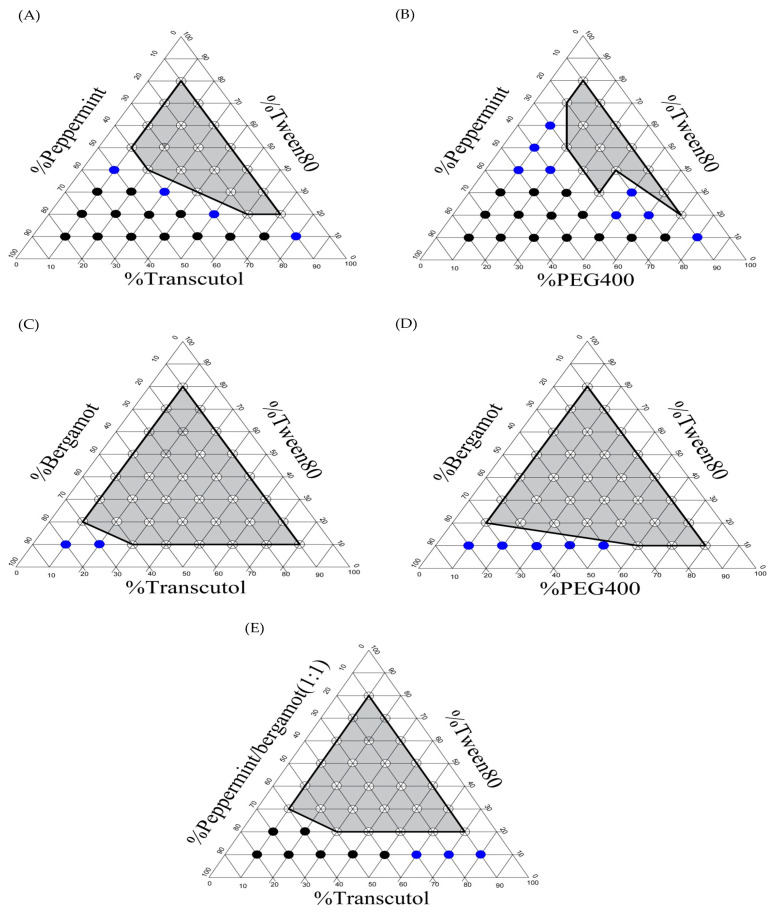
Ternary phase diagrams of different NE systems. (**A**) NE systems containing peppermint oil as an oil phase, and tween 80/transcutol P as S_mix_, (**B**) NE systems containing peppermint oil as an oil phase, and tween 80/PEG 400 as S_mix_, (**C**) NE systems containing bergamot oil as an oil phase, and tween 80/transcutol P as S_mix_, (**D**) NE systems containing bergamot oil as an oil phase, and tween 80/PEG 400 as S_mix_, and (**E**) NE systems containing peppermint/bergamot (1:1) oils as an oil phase, and tween 80/transcutol P as S_mix_. blank circles refer to clear NE points. Blue circles refer to bluish-white microemulsion points. Black circles refer to turbid macroemulsion points.

In terms of co-surfactant validation, transcutol P or PEG 400 were used in combination with tween 80, and the areas of the formed clear NE system were calculated. As shown in Figure 1, for both peppermint and bergamot oils, S_mix_ composed of tween 80/transcutol P exhibited a higher NE area compared to S_mix_ consisting of tween 80/PEG400. The areas of clear Nes prepared with S_mix_ composing of tween 80/transcutol P were 23% and 47% for peppermint and bergamot oil, respectively, which was remarkably higher than those prepared with S_mix_ composing of tween 80/PEG 400 (14% for peppermint oil and 44% for bergamot oil). Collectively, tween 80 and transcutol P were selected as the optimal S_mix_ for the formulation of the NE system.

### 2.3. Construction of Phase Diagrams

Ternary phase diagrams were created in order to determine the proper components and their concentration ranges that can result in a large NE existence area (Figure 1). As shown in Figure 1A,B, NE systems containing peppermint oil as an oil phase and tween 80/transcutol P or tween 80/PEG 400 as S_mix_ demonstrated a small and narrow NE region in the phase diagram. On the other hand, the NE system, which contains bergamot oil as an oil phase and tween 80/transcutol P or tween 80/PEG 400 as S_mix_, demonstrated a relatively larger NE region in the phase diagram (Figure 1C,D). Of note, despite the type of oil used, higher NE areas were observed when using transcutol as a co-surfactant rather than PEG 400. This might be attributed to the higher efficacy of short- to medium-chain-length alcohols (C3–C8) to minimize interfacial tension and endorse interface fluidity compared to their long-chain counterparts [41]. Interestingly, since GM shows the highest solubility in peppermint oil and tween 80 displays greater miscibility with bergamot oil, a mixture of peppermint oil/bergamot oil (1:1% *w*/*w*) was examined as the oil phase and tween 80/transcutol P as S_mix_. As shown in Figure 1E, this NE system demonstrated a relatively large NE area, and hence, it had been selected for the formulation of drug-loaded NE.

### 2.4. Characterization of GM-Loaded NEs

In this study, CCD was employed as a method to develop and optimize GM-loaded NEs. The 13 formulations given by the design were first evaluated for thermodynamic stability and the determination of the emulsion type, as shown below.

#### 2.4.1. Thermodynamic Stability Tests

Thermodynamic stability bestows the NE with a long shelf life compared to ordinary emulsions. In addition, it distinguishes NEs from kinetically stable emulsions, which usually suffer from phase separation. Accordingly, in this study, the thermodynamic stability of GM-loaded NEs was assessed following subsequent centrifugation, a heating and cooling cycle, and freeze–thaw cycles. All the tested formulations showed good thermodynamic stability in terms of their physical appearance and the absence of phase separation.

#### 2.4.2. Determination of Emulsion Type

The emulsion type of NEs was investigated by the dye test. The quick spreadability of all formulations, along with their homogeneous coloring with the hydrophilic methylene blue dye, affirms that the formulations were o/w type emulsions.

### 2.5. Statistical Analysis by CCD

The influence of two independent variables, namely oil concentration (A) and tween 80 concentration in the S_mix_ (B), on the three response variables, particle size (R_1_), zeta potential (R_2_), and DE% (R_3_), was evaluated in a total of 13 runs. The observed responses in the CCD of prepared NEs are depicted in Table 1. To assess the quantitative impacts of various formulation parameters at different levels on the dependent responses, the software generated polynomial equations for each response, which are the following:

Particle size (R_1_) = 131.24 + 144.65 A − 168.30 B − 62.54 AB + 77.69 A^2^ + 120.96 B^2^ Zeta potential (R_2_) = 7.19 − 1.77 A + 1.13 B + 0.245 AB + 1.36 A^2^ + 0.266 B^2^ DE% (R_3_) = 73.11 − 7.80 A + 10.51 B + 6.71 AB − 5.33 A^2^ − 4.95 B^2^

#### 2.5.1. Impact of Formulation Variables on Droplet Size

**Figure 2:**
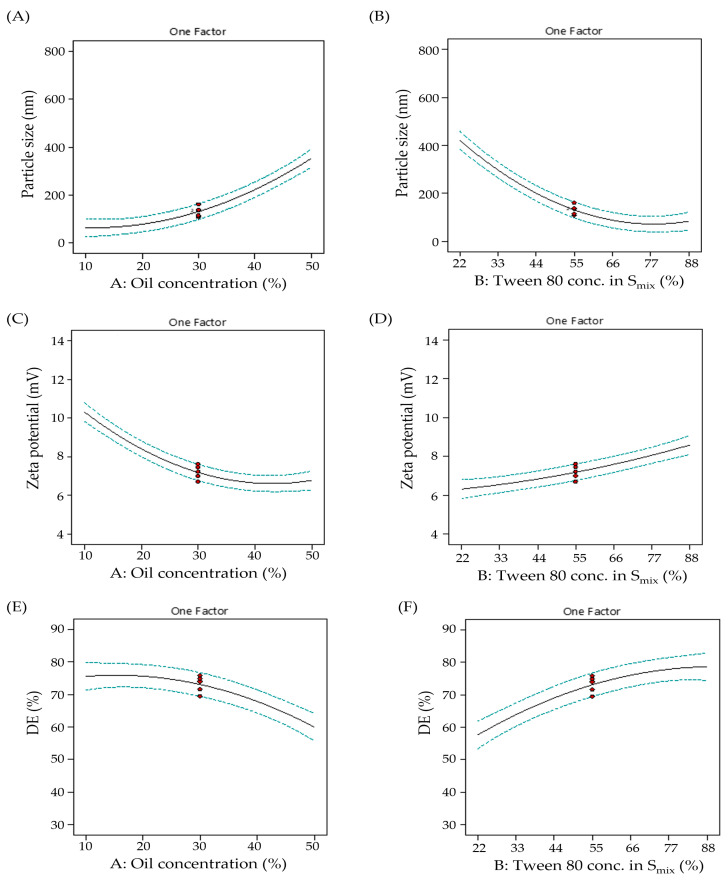
CCD plots showing influence of (**A**) Factor A on Y_1_ response (**B**) Factor B on Y_1_ response (**C**) Factor A on Y_2_ response (**D**) Factor B on Y_2_ response (**E**) Factor A on Y_3_ response (**F**) Factor B on Y_3_ response.

Droplet size has a great impact on the transdermal transportation of NEs. The smaller the droplet size, the higher the transdermal penetration of NE droplets. In this study, the droplet size of the developed NE formulations fluctuated between 66.0 ± 15.3 nm and 725.2 ± 18.0 nm (Table 1). In addition, it was evident that oil concentration (A) had a positive influence on the droplet size (Figure 2A), where increasing the oil concentration from 10% (F6) to 50% (F5) at fixed S_mix_ resulted in a significant increase in droplet size from 300.9 ± 25.4 nm to 725.2 ± 18 nm. This might be a result of globule expansion in droplets. On the other hand, rising tween 80 concentrations in the S_mix_ (B) resulted in a pronounced decrease in droplet size (Figure 2B). At a fixed oil concentration, increasing the tween 80 concentration in the S_mix_ (B) from 22% (F6) to 88% (F9), the droplet size remarkably decreased from 300.9 ± 25.4 nm to 150.8 ± 8.3 nm. It is well recognized that increasing the HLB value of surfactant decreases droplet size. Moreover, an increment in surfactant concentration could offer more surfactant accessible for adsorption and provide the formation of a stable, closely packed film of surfactant at the o/w interface, resulting in a smaller droplet size [42]. Accordingly, increasing the tween 80 concentration in the S_mix_ could afford a significant drop in droplet size.

#### 2.5.2. Impact of Formulation Variables on Zeta Potential

Zeta potential is an effective way of describing the surface potential of suspended droplets. Accordingly, the electrical characteristics of NE formulations were determined by measuring zeta potential values. All tested formulations exerted a negative zeta potential ranging from −5.81 ± 0.39 mV to −12.45 ± 0.26 mV (Table 1). NEs with negative surface charges are prone to electrostatic repulsion, which ensures a free-coalescence, well-separated emulsion system. It was evident that increasing oil concentration has a negative impact on droplet zeta potential (Figure 2C); increasing oil concentration decreased the surface charge on NE droplets. This effect might be attributed to the significant increase in droplet size upon increasing oil concentration, which in turn could lower the overall charge of dispersed NE droplets. On the other hand, as the tween 80 concentration increases, a significant decrease in droplet zeta potential is observed (Figure 2D). This happens because increasing the surfactant concentration over a certain amount might cause the abrupt expulsion of OH-groups from the o/w surface, which reduces the surface potential and therefore the zeta potential [43]. Nevertheless, tween 80 resulted in a modest increase in the negative charge at the o/w interface.

#### 2.5.3. Impact of Formulation Variables on DE%

The good release manner of drug-loaded NEs is crucial as it reflects the improved absorption of the drug followed by enhanced drug bioavailability in vivo. The studied NE formulations showed remarkable high releases of GM, with DE% values ranging from 35.92 ± 0.98% to 81.61 ± 1.28% (Table 1). It was clear that an increment in oil concentration resulted in a decrease in the release pattern of GM-loaded NE formulations and a decrease in their DE% values (Figure 2E). This could be attributed to the possible creation of larger-sized NE droplets as a result of increasing oil concentrations, exhibiting lessened surface area available to the medium [44]. In contrast, the impact of increasing tween 80 concentration on the DE% values of tested NEs was positively observed; increasing surfactant concentration increased the DE% values (Figure 2F). The instant NE formation due to the decreased droplet size could facilitate the enhancement of the rate and extent of drug dissolution, ensuring the ability of NEs to readily retain GM in solubilized form and positively influencing the GM bioavailability in vivo afterwards [45].

#### 2.5.4. Optimization Technique

The CCD-based numerical optimization was adopted to obtain an optimized NE formulation based on a preset number of constraints: minimum droplet size, maximum zeta potential, and DE%. The optimized formula for GM-loaded NE obtained at a desirability value of 0.892 was composed of 10% oil phase and a tween 80 concentration in the S_mix_ of 84.38%. The optimized formulation was fabricated for checkpoint analysis and assessed for droplet size, zeta potential, and DE%. The observed parameters were measured as 82.97 ± 12.62 nm, −11.87 ± 0.41 mV, and 76.21 ± 1.18%, respectively, which were comparable to the predicted values (66.03 nm, −11.32 mV, and 75.05%) for the optimized formula. These findings demonstrated the reliability of the optimization methodology for preparing NE formulations using the CCD.

### 2.6. Characterization of Optimized GM-Loaded NE Formulation

#### 2.6.1. TEM Study of the Optimized NE Formulation

**Figure 3:**
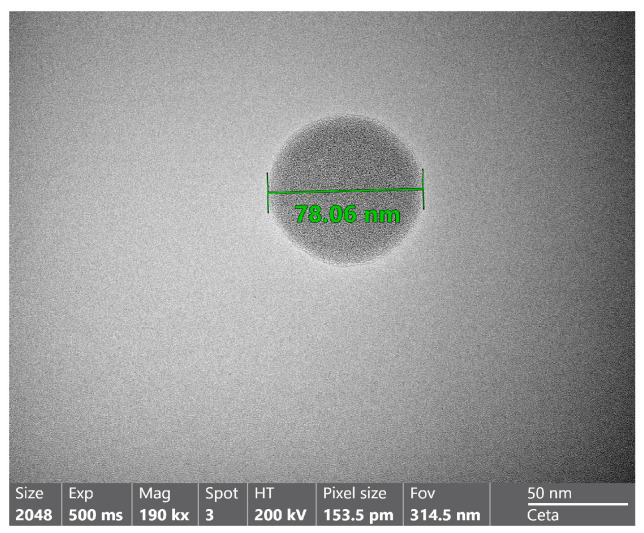
TEM image of optimized GM-loaded NE formulation.

TEM analysis was conducted to study the morphology of the optimized NE formulation. As shown in Figure 3, the spherical droplets of the NE appeared dark while the surroundings were bright. Ullah et al. [46] reported that the spherical shape of droplets is beneficial in facilitating the permeation of drug-loaded systems through the tight pores of the skin. In addition, droplet size measurements of the optimized NE formulation were conducted by TEM. The estimated droplet size of optimized NE was comparable to that obtained from the dynamic light scattering technique.

#### 2.6.2. DSC Study of the Optimized NE Formulation

**Figure 4:**
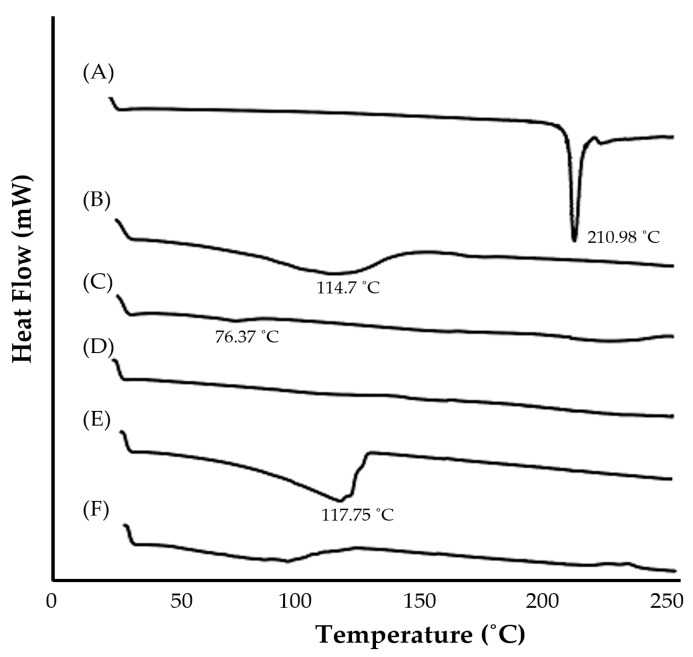
DSC thermograms of (A) pure GM; (B) peppermint oil; (C) bergamot oil; (D) tween 80; (E) transcutol P; and (F) optimized GM-loaded NE formulation.

In order to address the possible drug-excipient interactions, a DSC study was carried out. Figure 4 depicts DSC thermograms of pure GM, individual NE components, and an optimized NE formulation. The DSC thermogram of pure GM exhibited a sharp endothermic peak at 210.98 °C, which corresponds to its melting point, indicating its crystalline nature. The DSC thermograms of peppermint oil, bergamot oil, and transcutol P showed endothermic peaks at 114.7 °C, 76.37 °C, and 117.75 °C, respectively, while tween 80 showed no characteristic peak (Figure 4). Of interest, the DSC thermogram of drug-loaded NE formulations did not exhibit the characteristic endothermic peak of GM, suggesting the molecular dispersion and/or the increased solubility of GM in the oily phase of NE.

#### 2.6.3. FTIR Study of the Optimized NE Formulation

**Figure 5:**
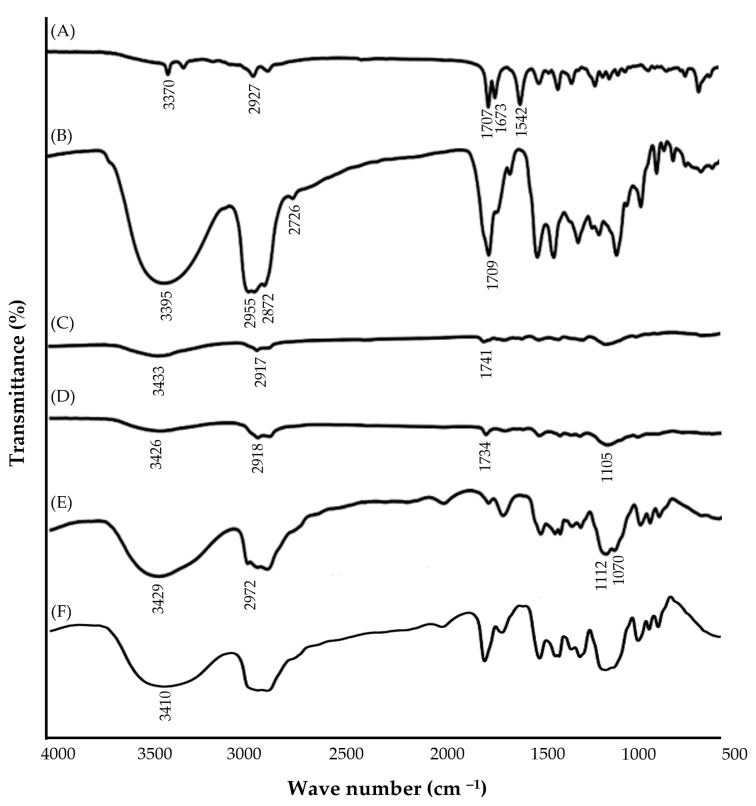
FTIR spectra of (A) pure GM; (B) peppermint oil; (C) bergamot oil; (D) tween 80; (E) transcutol P; and (F) optimized GM-loaded NE formulation.

The FTIR spectra of pure GM, NE components, and optimized NE formulations are depicted in Figure 5. The spectrum of pure GM revealed the presence of characteristic absorption peaks at 3370 cm^−1^ (N–H, stretching), 2927 cm^−1^ (C–H, stretching), 1707 cm^−1^ (C=O, stretching), and at 1673 and 1542 cm^−1^ (aromatic C=C). The FTIR spectrum of peppermint oil showed characteristic peaks at 3395 cm^−1^, 2955 cm^−1^, 2872 cm^−1^, 2726 cm^−1^, and 1709 cm^−1^, corresponding to the O–H stretching bond, the C–H stretching bond, aldehydic H–C=O, and the C=O stretching bond, respectively. Similarly, bergamot oil spectrum depicted characteristic peaks at 3433 cm^−1^, 2917 cm^−1^, and 1741 cm^−1^ referring to the O-H stretching bond, the C–H stretching bond, and the C=O stretching bond, respectively. Tween 80 exhibited stretching peaks for O–H bonding (3426 cm^−1^), C–H aliphatic vibration (2918 cm^−1^), carbonyl vibrations (1734 cm^−1^), and C–O vibrations (1105 cm^−1^). Transcutol P spectra showed stretching peaks for O–H bonding (3429 cm^−1^), C–H aliphatic vibration (2972 cm^−1^), and C–O vibrations (1112 cm^−1^ and 1070 cm^−1^). Of interest, for GM-loaded NE formulations, the most characteristic peaks of GM, incorporated in NE, were still present even with lower intensity, presumably due to overlapping with other peaks of NE ingredients. These results suggest the absence of any remarkable interaction between GM and NE ingredients. 

### 2.7. In-Vitro Release Study of the Optimized NE Formulation

**Figure 6:**
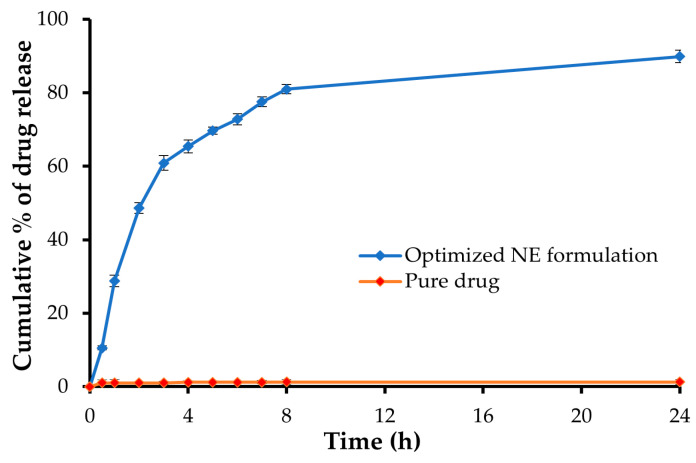
In-vitro release of GM from optimized GM-loaded NE formulation.

Figure 6 depicts the in vitro release of pure GM and GM from an optimized NE formulation. Due to its limited water solubility, less than 5% of the pure drug was released after 24 h. On the other hand, the optimized NE formulation demonstrated a significantly higher drug release rate (*p* < 0.05), with about 90% of the loaded drug released at the end of the release study (at 24 h). Such enhanced drug release from optimized NE formulation might be attributed, on the one hand, to the tiny globule size with the increased surface area exposed to the release medium and, on the other hand, to the high polarity of NE formulation due to the right balance of the oil:S_mix_ ratio, which exerts a potent solubilization power for the loaded drug [47].

### 2.8. Preparation of GM-Loaded Nanoemulgel Formulations

Hydrogel systems can provide excellent homogeneity with colloidal systems and improve lipophilic drug penetration, making them appealing transdermal dosage forms in the pharmaceutical area. In this study, the optimized drug-loaded NE formulation was incorporated into different gel bases, namely carbopol 940, carbopol 934, Na-CMC, sodium alginate, or HPMC. The prepared nanoemulgel formulations were evaluated for gel strength, and formulations with an acceptable gel strength of 25–50 s were selected for further investigations. As shown by the preliminary study (Table S3), the gel strength values remarkably increased by raising the gel:NE ratio. This might be attributed to the increased cross-linking densities achieved by increasing the gel fraction, which results in improved structural integrity of the gel structure [48]. Gel formulations with gel strength less than 25 s are known to exert weak gel behavior with a high tendency to leak away from the skin after application [49]. Accordingly, nanoemulgel formulations with gel strength values less than 25 s were excluded from further investigations.

### 2.9. Characterization of GM-Loaded Nanoemulgel Formulations

#### 2.9.1. Visual Inspection

Visual inspection of different nanoemulgel formulations for homogeneity, transparency, and grittiness verified the ideal characteristics for topical nanoemulgel formulations of being homogenous, clear, and free from undissolved drug particles.

#### 2.9.2. pH Measurement

The pH values of different nanoemulgel formulations are summarized in Table 2. The pH values fluctuate from 5.46 ± 0.08 (G4) to 6.36 ± 0.11 (G9). The normal pH of human skin ranges from 4 to 6.5 [50]. Consequentially, all the prepared nanoemulgel formulations seem to be non-irritant to the skin following topical application.

#### 2.9.3. Viscosity Determination

Viscosity not only influences features such as spreadability and skin feel but also affects the skin penetration of incorporated actives. Viscosity values obtained for various nanoemulgel formulations are summarized in Table 2. It was evident that the developed nanoemugel formulations had low viscosity values ranging from 9800 ± 163.29 cP (G4) to 14266.67 ± 205.48 cP (G8), indicating that they had a suitable consistency to be applied to the skin [16].

#### 2.9.4. Spreadability Determination

Spreadability refers to the ability of a gel to spread over an area upon application to the skin surface. It represents a viable characteristic of topical formulation for uniform gel application onto the skin surface and better patient compliance [34]. Generally, high spreadability values would ease the topical application of gel formulations onto the skin surface. Herein, the spreadability values were found to be in the range of 1.13 ± 0.12 (G7) to 5.25 ± 0.15 cm (G4) (Table 2), depending on the gel base used. In addition, gel spreadability was inversely proportional to the viscosity of different nanoemulgel formulations.

### 2.10. In-Vitro Release Study

**Figure 7:**
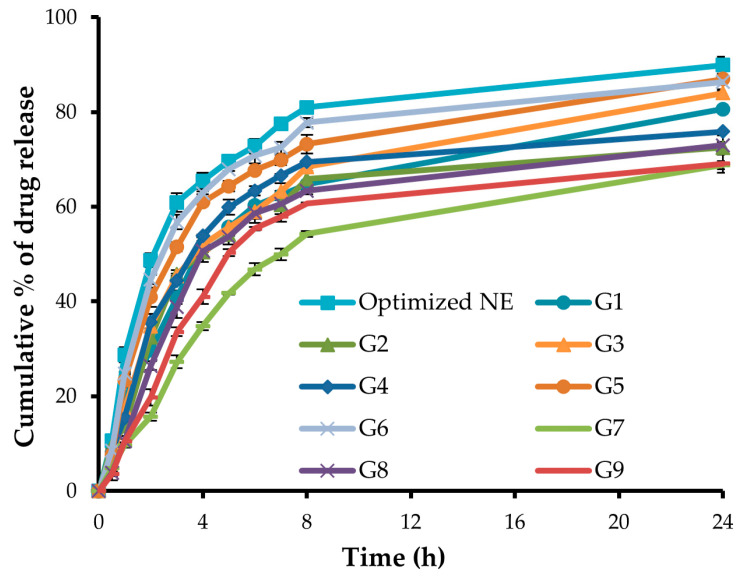
In-vitro drug release from different GM-loaded nanoemulgel formulations.

Dissolution studies were conducted using a dialysis membrane to compare the release pattern of GM from the nine different nanoemulgel formulations (G1–G9) against the optimized drug-loaded NE. As depicted in Figure 7, the in vitro release of GM from different nanoemulgel formulations was remarkably lower than that from the NE formulation. The slower drug release from nanoemulgel formulations might be attributed to the fact that, in nanoemulgel formulations, the drug-loaded oil droplets are covered by the polymeric surface; consequently, the drug must travel a longer pathway to reach the release medium, resulting in lower drug release. Nonetheless, incorporation of GM-loaded NE in gel base did not adversely hinder drug release, where more than 75% of loaded drug was released from different gel formulations at 24 h.

Importantly, DE%, the area under the dissolution curve up to a specified time, was calculated for different formulations and adopted as a selection criterion for optimized nanoemulgel formulations. As summarized in Table 2, among various nanoemulgel formulations, formula G6, composed of carbopol 934 (2%) at a gel:NE ratio of 1.25:1, showed the highest DE% (72.91 ± 0.98%), and accordingly was selected for further ex vivo and in vivo investigations.

### 2.11. Ex-Vivo Permeability Study of the Optimized Nanoemulgel

**Figure 8:**
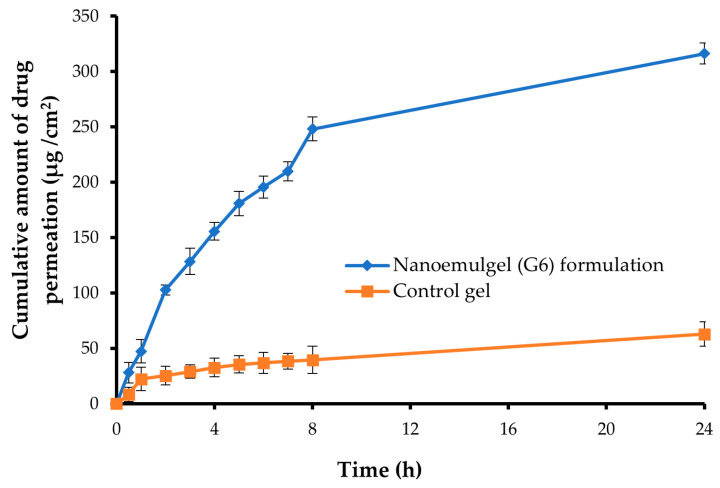
Ex-vivo permeation of GM from either control GM gel or GM-loaded nanoemulgel formulation (G6).

The ex-vivo permeability of GM across abdominal rabbit skin from optimized GM-loaded nanoemulgel or GM-loaded control gel was examined as an indicator for predicting the overall in-vivo behavior. As shown in Figure 8, loading GM within the nanoemulgel formulation (G6) significantly boosted skin permeation of the loaded drug when compared with the control gel (*p* < 0.001). The cumulative amounts of drug permeated through the skin from optimized GM-loaded nanoemulgel or GM-loaded control gel were 316.21 ± 9.63 μg/cm^2^ and 62.75 ± 10.85 μg/cm^2^, respectively, at 24 h. This superior permeation of GM-loaded nanoemulgel might be attributed, on the one hand, to the nanosized globules of the formulation, which could enhance drug solubilization and the surface area available for drug permeation, and, on the other hand, to the ability of formulation components to disrupt the lipidic barrier of the skin surface [51]. Several studies have emphasized the penetration-enhancing effect of tween 80 on the percutaneous absorption of topically applied drugs [52,53]. Furthermore, transcutol P was acknowledged for its efficacy to facilitate drug penetration through biological membranes by being integrated within the membrane lipid bilayers and altering their structure, which in turn would enhance drug permeability [54].

The values of the permeation parameters for both optimized GM-loaded nanoemulgel and GM-loaded control gel are listed in Table 3. The J_ss_ of GM from the optimized nanoemulgel formulation was 29.20 ± 4.85 μg/cm^2^·h^−1^, which was significantly higher than that from naïve GM-loaded gel (*p* < 0.001). Furthermore, drug permeation from the optimized GM-loaded nanoemulgel was enhanced by 6.90 folds compared to naïve GM-loaded gel. These results collectively confirm the feasibility of nanoemulgel formulation in promoting efficient skin penetration of loaded drugs into deep layers of skin, which could help in potentiating their anti-diabetic effect.

### 2.12. Histopathological Study of the Optimized Nanoemulgel

**Figure 9:**
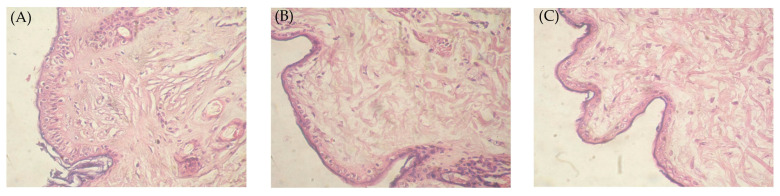
Histopathological study of (**A**) control skin sample; (**B**) skin sample treated with control GM gel; and (**C**) skin sample treated with optimized GM-loaded nanoemulgel formulation (G6).

In order to assess the safety of an optimized drug-loaded nanoemulgel formulation after topical application, histopathological studies were conducted. Rabbit skin samples treated with either the optimized GM-loaded nanoemulgel formulation (G6) or the control gel formulation were compared to a saline-treated normal skin sample (Figure 9). The stained normal skin revealed a well-defined structure with epidermis, dermis, and subcutaneous layers, as well as normal skin appendages and vascularity (Figure 9A). Of interest, no signs of inflammation and/or irritation were observed in stained skin samples treated with either control gel (Figure 9B) or optimized nanoemulgel (Figure 9C). Furthermore, no alterations in skin structure were observed upon topical application of either the GM-loaded nanoemulgel formulation or the control gel formulation. These results clearly verify the safety/tolerability of drug-loaded nanoemulgel formulations.

### 2.13. In-Vivo Pharmacokinetic Study of the Optimized Nanoemulgel

**Figure 10:**
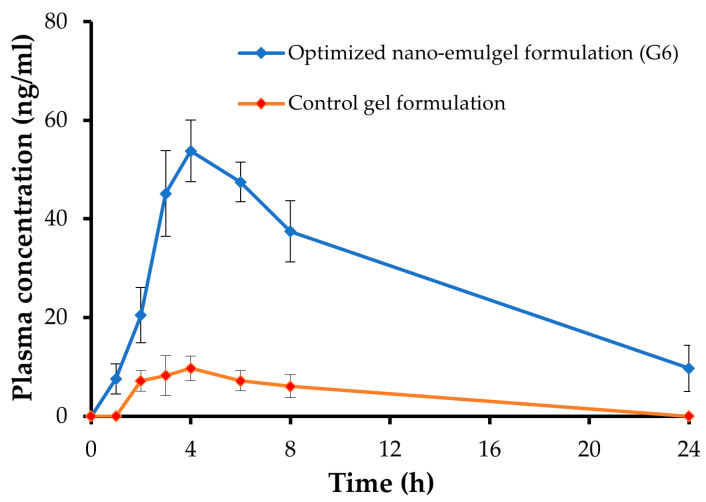
Plasma concentration-time curve of GM following topical application of either control GM gel or optimized GM-loaded nanoemulgel formulation.

Figure 10 illustrates the blood concentration-time curve of GM following topical application of the optimized GM-loaded nanoemulgel formulation (G6) and control GM gel. It was evident that the optimized GM-loaded nanoemulgel formulation had higher GM plasma concentrations than the control GM gel. The C_max_ of GM following topical application of the optimized nanoemulgel formulation was 53.75 ± 6.27 ng/mL, which was significantly higher than that following application of the control gel (9.68 ± 2.51 ng/mL). The higher C_max_ of the optimized nanoemulgel formulation compared to the control GM gel might be attributed to the enhanced transdermal permeability of the optimized nanoemulgel formulation.

The pharmacokinetic parameters are illustrated in Table 4. The AUC_0–24h_ for optimized GM-loaded nanoemulgel formulation was 666.89 ± 36.27 ng/mL·h, which was substantially higher than control GM gel (61.00 ± 19.35 ng/mL·h) (*p* < 0.05). In addition, compared to the control GM gel, the GM-loaded nanoemulgel formulation efficiently extended the MRT of GM in the systemic circulation. The MRTs for the optimized GM-loaded nanoemulgel formulation and control gel were 12.36 ± 2.44 h and 9.69 ± 2.03 h, respectively. Interestingly, incorporation of GM into nanoemulgels increased GM systemic bioavailability substantially. The GM-loaded nanoemulgel formulation showed a 10-fold increase in relative bioavailability compared to the control GM gel. The substantial increment in the systemic bioavailability of the GM-loaded nanoemulgel formulation compared to the control gel might be ascribed, at least in part, to the reduction of the droplet size in the formulation, which would facilitate drug penetration via outer skin barriers. Furthermore, the ability of formulation components (tween 80 and transcutol P) to disrupt the lipidic barrier of the skin surface could substantially contribute to the enhancement of transdermal permeability of the formulation [53,54].

## 3. Conclusions

In this study, the hypoglycemic drug GM was successfully incorporated into a NE system consisting of peppermint/bergamot oil as the oily phase, tween 80 as a surfactant, and transcutol P as a co-surfactant. The formulated NE systems were optimized using the CCD. The optimized GM-loaded NE formulation showed a small spherical droplet with a reasonable zeta potential and efficiently enhanced in-vitro GM release compared to the pure drug. In addition, the optimized GM-NE formula was then incorporated into different gel bases. The prepared nanoemulgel formulations exhibited good physical properties and considerably boosted the transdermal flux of GM across abdominal rabbit skin compared to plain GM gel. Most notably, in vivo pharmacokinetic studies underscored the efficacy of optimized nanoemulgel formulation to augment the systemic bioavailability of GM by promoting a 10-fold increase in AUC_0–24h_, compared to control GM gel. To sum up, NE might be a plausible carrier for upgrading transdermal GM delivery and augmenting its hypoglycemic activity.

## 4. Materials and Methods

### 4.1. Materials

GM was obtained from Delta Pharma Co. (10th of Ramadan, Egypt). Peppermint, bergamot, mandarin, tea tree, eucalyptus, fennel, lavender, rosemary, orange, and lemon oils were provided by Nefertari Natural Body Care Line (Cairo, Egypt). Labrafil isostearic, labrafil M 1944 CS, transcutol P, and lauroglycol FCC were kindly gifted from Gattefossé Co. (Saint-Priest, France). Tween 80, propylene glycol (PG), ethanol, and glycerin were purchased from El-Nasr Pharmaceutical Chemicals (Cairo, Egypt). Tween 20 was purchased from Oxford Lab Chem (Vasai, India). Span 20 and Span 85 were purchased from Sigma Chemical Co., St. Louis, MO, USA. Polyethylene glycol 400 (PEG 400) was procured from Fluka Chemie AG (Buchs, Switzerland). Carbopol 934, carbopol 940, sodium carboxymethyl cellulose (Na-CMC), sodium alginate, and hydroxypropyl methyl cellulose (HPMC) were provided by EIPICO Co. (10th of Ramadan, Egypt).

### 4.2. Solubility Studies

Excess amounts of GM were mixed with 3 mL of various oils, surfactants, and co-surfactants in sealed glass vials and placed in a thermos-balanced shaker water bath for 72 h at 25 ± 1 °C. The samples were filtered using 0.22 µm syringe filter membranes after being centrifuged for 15 min at 10,000× *g* rpm. The GM concentration in the filtrates was spectrophotometrically assessed at λ_max_ 227 nm. Peppermint, bergamot, mandarin, tea tree, eucalyptus, fennel, lavender, rosemary, orange, and lemon oils were the oils that were studied. Span 20, span 85, tween 20, tween 80, labrafil M 1944 CS, and labrafil isostearic were examined as surfactants, while ethanol, PG, lauroglycol FCC, PEG 400, transcutol P, and glycerin were the investigated co-surfactants.

### 4.3. NE components Screening and Selection

#### 4.3.1. Surfactant Validation

The surfactants showing higher GM solubilization capacity were examined for their miscibility with the greatest concentration of oils in which the drug was highly solubilized. Briefly, fixed amounts of the chosen oils (5 µL) were repeatedly added to a fixed volume (2.5 mL) of surfactant aqueous medium (15% *w*/*v*) with vigorous agitation until the clear solution turned turbid [42].

#### 4.3.2. Co-Surfactant Validation

Using a variety of co-surfactants, ternary phase diagrams were constructed to show the maximum solubilization capacity of GM, which was used to confirm the composition of NE. The co-surfactants were plotted against the selected oils and surfactants using the AUTODESK^®^AUTOCAD^®^ program, version 22. Each point in these ternary phase diagrams was developed and visually reviewed for the creation of a distinct one-phase solution after being diluted with a constant 100 mL of distilled water using a magnetic stirrer. The evaluation criterion for choosing a co-surfactant was to compare the extent of the clear NE regions within phase diagrams [39]. The point showing a rapid, clear appearance after dilution with water was classified as a self-NE. The point with a bluish-white appearance was graded as a self-microemulsion, while the point with a milky-turbid appearance was graded as a macroemulsion. The preparations that represented the self-NE appearance were selected for further study using the CCD.

### 4.4. Experimental Design of GM-Loaded NEs

The impact of the independent factors, oil concentration (A) and tween 80 concentration in the surfactant/co-surfactant mixture (S_mix_) (B), on the three response variables, particle size (R_1_), zeta potential (R_2_), and dissolution efficacy (DE%) (R_3_), was examined using a two-factor, five-level CCD (Design-Expert^®^ software, version 11). Table 5 demonstrates the goal criteria for the measured responses and the lower and upper limits of the independent factors. Thirteen investigations were developed from the CCD, as shown in Table 1. The experimental design consisted of five center points, four factorial points, and four axial points, and it was conducted in a random manner. To assess the reproducibility of the implemented approach, the center point was replicated five times. The response surface regression approach was used to examine the data. A quadratic model was selected depending on the non-significant lack of fit and the significant parameters (*p* < 0.05) generated by the software.

For evaluating the best fitness of the data, comparisons between several statistical parameters were made, and the relations between the independent factors and the response variables were studied. Following the statistical analysis, an optimized NE formulation was chosen using the optimization technique depending on the goals listed in Table 1. The experimental values of the optimum NE were compared with those anticipated by the software design to verify the validity of the design.

### 4.5. Preparation of GM-Loaded NEs

The spontaneous emulsification approach was used to prepare GM-loaded NEs [55]. A definite amount of GM (2 mg) was dissolved in selected oils to form the oily phase. GM-loaded NEs were then created by mixing the oily phase and S_mix_ at the optimum ratios derived from the phase diagram studies using a vortex mixer.

### 4.6. Characterization of GM-Loaded NEs

#### 4.6.1. Thermodynamic Stability Studies

Through a three-step approach, the formulations’ thermodynamic stability was confirmed. Beginning with a 30-min centrifugation at 15,000× *g* rpm, samples were examined for any indication of phase separation, creaming, or cracking before being discarded. To ascertain the impact of temperature fluctuations on their stability, samples were then kept under six consecutive cycles of heating (45 °C) and cooling (4 °C) for not less than 48 h at each temperature. In order to determine the formulations’ effective dispersibility, three freeze-thaw cycles between 20 °C and 25 °C were performed, with storage at each temperature for 48 h.

#### 4.6.2. Determination of Particle Size and Zeta Potential of NEs

A Malvern Zeta-sizer (Nano–ZS90, Malvern Instruments Ltd., Malvern, UK) was used to expose the samples to the dynamic light scattering technique. In order to reduce the impact of multiple scattering, samples were diluted with distilled water (1:100). The tested formulations’ results for particle size and zeta potential were recorded.

#### 4.6.3. In-Vitro Drug Release and DE% Measurement

The in vitro release of the tested formulae was assessed using a thermo-balanced shaker water bath maintained at 37 ± 0.5 °C and 50 rpm. Briefly, a definite volume of nanoemulsion formulations (2 mg) of GM was kept in a dialysis bag suspended in 100 milliliters of receptor media (phosphate buffered saline, pH 5.5). At various time intervals, aliquot samples (2 mL) were withdrawn and replaced by an equal volume of receptor media to maintain sink conditions. The samples were filtered, diluted, and analyzed for drug concentration using a UV-Vis spectrophotometer at λ_max_ 227 nm. The release profiles were evaluated based on mean DE% values. The DE% was assessed using the trapezoidal rule by the Microsoft Excel add-in DDsolver program from the area under the dissolution curve for 24 h. This value was expressed as a percent of the rectangle area described by 100% dissolution at the same time [56].

#### 4.6.4. Transmission Electron Microscopy of the Optimum GM-Loaded Nanoemulsion

The prepared sample was put directly onto a 200-mesh carbon-coated copper grid and diluted with water (1:10) before being studied at 200 kV with a transmission electron microscope.

#### 4.6.5. Differential Scanning Colorimetry (DSC) of the Optimized GM-Loaded NE

Using DSC equipment (DSC W70, Shimadzu, Kyoto, Japan), the DSC investigation of pure GM, oils, surfactant, co-surfactant, and optimized GM-loaded NE was carried out. Each sample, weighing approximately 5 mg, was sealed in a conventional DSC aluminum pan and heated at a rate of 10 °C/min before being scanned over a temperature range of 25 to 250 °C under nitrogen purge (30 mL/min) [57].

#### 4.6.6. Fourier Transform Infrared (FTIR) Spectroscopy of the Optimized GM-Loaded NE

Utilizing a Perkin-Elmer FTIR spectrophotometer (series 1600, Perkin-Elmer Corporation, Norwalk, CT, USA) and the potassium bromide disk method, the FTIR spectra of pure GM, oils, surfactant, co-surfactant, and optimized GM-loaded NE were recorded [58].

### 4.7. Formulation of GM-Loaded Nanoemulgels

Nanoemulgel formulations loaded with GM were prepared using different gelling agents, namely carbopol 940, carbopol 934, Na-CMC, sodium alginate, and HPMC, at different concentrations. Concerning the formulation using carbopol 940 and 934 gel bases, an appropriate quantity of each gelling agent (1–2% *w*/*w*) was soaked in distilled water for two hours and mixed using a magnetic stirrer. Then, a specified amount of the optimized GM-loaded NE formulation, equivalent to 2 mg of drug, was added dropwise at various ratios to the prepared polymeric gels and mixed for twenty additional minutes. The dispersion was hydrated and swelled for sixty minutes, and the pH was adjusted with triethanolamine to a pH range of 5.5–6 [59].

Other nanoemulgel formulae were generated by dispersing 5% *w*/*w* Na-CMC, 5% *w/w* sodium alginate, or 5% *w*/*w* HPMC in distilled water with continuous stirring for 2 h. A specified amount of the optimized GM-loaded NE formulation, equivalent to 2 mg of drug, was added individually to each gelling solution at different ratios under continuous stirring. The mixture was gently mixed with a spatula until a homogeneous gel was obtained. All mixtures were equilibrated for at least 24 h in the refrigerator prior to performing further investigations [60]. The preliminary study included the measurement of gel strength of various gel nanoemulgel formulations (Table S3), and those that displayed satisfactory values were examined for their viscosity, spreadability, and drug release studies.

### 4.8. Characterization of GM-Loaded Nanoemulgels

#### 4.8.1. Visual Assessment for Clarity and pH Measurement

The created formulas were examined against a white background for color, grittiness, and clarity. Glass slides were covered with formulation smears, which were then examined for the presence of any grittiness, aggregates, or insoluble particles. By dipping the glass electrode of a digital pH meter into a 50 mL beaker holding an adequate amount of each manufactured formulation at room temperature, the pH values of the tested nanoemulgel formulations were ascertained [61].

#### 4.8.2. Gel Strength Measurement

Gel strength was estimated as the number of seconds needed for a weight of 3.5 gm to pass through 5 gm of gel placed in a graduated measuring cylinder to descend 3 cm [60]. As shown in Table S3, the gel formulations that demonstrated acceptable gel strength values were subjected to further investigations.

#### 4.8.3. Viscosity Measurement

The viscosity of the preparations (G1–G9) was determined using a viscometer (Visco Star-R, Fungilab S.A., Barcelona, Spain) at 25 ± 1 °C and 10 rpm with spindle no. 6 [62].

#### 4.8.4. Spreadability Measurement

Briefly, a circle with a 1 cm diameter was created on a glass plate, and 1 g of each nanoemulgel formulation (G1–G9) was placed inside. A second glass plate was then placed on top of the first one, and for 5 min, a specified weight (500 gm) was placed on the top plate to stop the spreading of the gel compositions. The spreadability value was calculated as an increase in the gels’ circle diameter [63].

### 4.9. In-Vitro Drug Release and DE% Measurement

The in vitro release of the tested formulations (G1–G9) was performed using a thermo-balanced shaker water bath, maintained at a temperature of 37 ± 0.5 °C and 50 rpm. Briefly, a definite weight of nanoemulgel formulations (equivalent to 2 mg of GM) was kept in a dialysis bag suspended in 100 mL of receptor media (phosphate buffered saline, pH 5.5). 2 mL samples were taken out at regular intervals and replaced with equal quantities of the new release medium. The samples were analyzed for drug concentration using a UV-Vis spectrophotometer at λ_max_ 227 nm. The cumulative percentage of GM released was blotted versus time, and the in-vitro release profiles were evaluated based on the mean DE% values as above mentioned. The optimum GM-loaded nanoemulgel formulation was selected for further ex-vivo and in-vivo evaluations according to its acceptable gelling structure, in addition to the closeness of its in-vitro release profile and DE% value to those of the optimized GM-loaded NE formulation.

### 4.10. Ex-Vivo Drug Permeation Study of the Optimized Nanoemulgel

The ex vivo permeation of the optimum GM-loaded nanoemulgel formulation was studied utilizing fresh abdomen skin taken from the sacrificed rabbit and brought from the local slaughterhouse right away. In the donor compartment of a diffusion cell, a specific weight of each formulation (equal to 2 mg GM) was added. Hundred milliliters of phosphate buffered saline (pH 5.5) and sodium azide (0.02%) as a preservative were both present in the receiver chamber. To maintain the sink condition, 2 mL samples were taken out at intervals of 0, 0.5, 1, 2, 3, 4, 5, 6, 7, and 24 h and replaced with equal quantities of new release medium. Spectrophotometric detection of the drug content was performed at 227 nm. Permeation analysis was performed according to the following equations [64]:

$$J_{ss}=\frac{Slope\;of\;the\;linear\;part\;of\;graph}{area\;of\;diffusion\;cell}$$

$$K_{p}=\frac{J_{ss}}{C_{o}}$$

$$Er=\frac{J_{ss(formulation)}}{J_{ss(control)}}$$

where, C_o_ is the initial drug concentration, K_p_ is the permeability coefficient, J_ss_ is the steady state drug flux, and Er is the enhancement ratio.

### 4.11. Histopathological Study of the Optimized Nanoemulgel

Three uniformly sized samples of abdominal rabbit skin were examined. A half-milliliter of saline was applied to a fresh skin portion (negative control). The second and third samples treated with the control gel and the optimum nanoemulgel formulations, respectively, were taken after the completion of the ex-vivo permeation study. The skin was washed with phosphate buffered saline (pH 5.5) after one hour and then left in neutral formalin (10%) overnight. After being cut vertically, the skin samples were dehydrated using ethanol before being embedded in paraffin blocks. Hematoxylin and eosin were used to stain the microtome-taken fine sections. The stained sections were finally visualized under a polarizing microscope (Radical Scientific Equipment Pvt. Ltd., Mumbai, India) equipped with a Nikon Optiphot camera.

### 4.12. In-Vivo Characterization of the Optimized Nanoemulgel

#### 4.12.1. Ethical Approval

The animal breeding facility at Zagazig University in Egypt provided adult albino male rats weighing 250–300 gm each. Before the experiments, the animals were kept at room temperature for a week in a 12-h light, 12-h dark cycle with access to free food and water. The studies were carried out in accordance with the recommendations of the Faculty of Pharmacy’s Institutional Animal Care and Use Committee (IACUC; ZU-IACUC/3/F/120/2022).

#### 4.12.2. In-Vivo Pharmacokinetic Study

The animals were divided randomly into two groups (n = 6); Group 1: rats received the control gel formulation (10 mg GM/kg) and Group 2: rats received the optimum nanoemulgel formulation (10 mg GM/kg) [13]. At various times (0, 1, 2, 3, 4, 6, 8, and 24 h), blood samples were taken from the lateral tail vein of animals and placed into heparinized tubes. The samples were immediately centrifuged for 10 min at 3000× *g* rpm. They were then kept at −20 °C. For drug extraction, each plasma sample (0.1 mL) was added to 0.5 mL chloroform and vortexed for 5 min using a vortex mixer (Purimix, Cryste-Novapro, Bucheon, Korea). It was then centrifuged at 5000× *g* rpm for 10 min using a centrifuge. The supernatants were evaporated under a nitrogen stream (109A00126WP, Glas-Col, Terre Haute, IN, USA), reconstituted with 0.12 mL of mobile phase, and filtered using a 0.22 μm syringe filter. Then, 100 μL was injected into the HPLC system for analysis. HPLC analysis was conducted using an HPLC system (2690 Waters, Milford, Godalming, UK) equipped with an X-Terra C18 column (4.6 × 100 mm, 5 μm). Elution pumps ran a modified isocratic mobile phase consisting of distilled water:acetonitrile (50:50% *v*/*v*). The flow rate was 1 mL/min. The HPLC assay was carried out at 227 nm using a photodiode array detector. The calibration curve was used to determine the drug concentration in the plasma samples.

A non-compartmental method was utilized to conduct the pharmacokinetic calculations (Microsoft Excel add-in PKsolver program, version 2). Directly from the individual plasma concentration-time profiles, the maximum plasma concentration (C_max_) and the time needed to achieve the C_max_ (t_max_) were determined. The terminal slope of each plasma concentration-time curve was used to calculate the apparent elimination rate constant (K_el_). The trapezoidal technique was used to determine the areas under the plasma concentration-time curves (AUC_0–24h_ and AUC_0–∞_). Calculations were made for the elimination half-life (t_1/2_), mean residence time (MRT), and relative bioavailability. The pharmacokinetic parameters that were obtained were analyzed using the Student’s *t* test. Version 5 of the GraphPad Prism^®^ software was employed.

## Figures and Tables

**Table 1:** Composition of GM-loaded NE and their physicochemical characteristics.

Formula	A: Oil Concentration (%)	B: Tween 80 Conc. in S-Mix (%)	Particle Size (nm)	Zeta Potential (mV)	DE (%)
F1	30	55	137.3 ± 7.2	−6.69 ± 0.39	74.83 ± 1.34
F2	50	88	279.9 ± 18.3	−8.70 ± 0.73	67.64 ± 1.63
F3	30	101.66	100.5 ± 8.9	−9.24 ± 0.54	81.61 ± 1.28
F4	30	55	108.3 ± 8.2	−7.45 ± 1.02	75.75 ± 1.18
F5	50	22	725.2 ± 18.0	−6.11 ± 0.73	35.92 ± 0.98
F6	10	22	300.9 ± 25.4	−9.80 ± 0.57	68.23 ± 1.12
F7	30	55	135.9 ± 11.0	−6.99 ± 0.71	73.97 ± 1.32
F8	30	8.33	599.7 ± 14.5	−5.81 ± 0.89	48.02 ± 1.05
F9	10	88	105.8 ± 8.3	−11.41 ± 0.30	73.12 ± 0.79
F10	58.28	55	461.1 ± 24.1	−6.96 ± 0.23	55.34 ±1.47
F11	30	55	114.1 ± 10.1	−7.21 ± 0.72	71.59 ± 1.49
F12	30	55	160.5 ± 7.5	−7.60 ± 0.78	69.42 ± 0.65
F13	1.71	55	66.0 ± 15.3	−12.45 ± 0.26	72.75 ± 1.71

**Table 2:** Physicochemical characterization of different GM-loaded nanoemulgel formulations.

Formula	Gel Base Type	Gel:NE Ratio	Gel Strength (s)	pH	Viscosity (cP)	Spreadability (cm)	DE (%)
G1	Carbopol 940 (1%)	2.5:1	28.13 ± 0.84	5.55 ± 0.03	12,666.7 ± 262.5	3.97 ± 0.09	63.09 ± 0.79
G2	Carbopol 940 (1.5%)	1.5:1	26.30 ± 1.02	5.52 ± 0.08	10,133.3 ± 124.7	4.30 ± 0.08	60.68 ± 1.52
G3	Carbopol 940 (2%)	1:1	26.25 ± 0.91	5.62 ± 0.06	10,300.0 ± 216.0	4.67 ± 0.12	65.81 ± 1.07
G4	Carbopol 934 (1%)	3.5:1	25.26 ± 0.86	5.46 ± 0.08	9800.0 ± 163.3	5.25 ± 0.15	64.01 ± 0.83
G5	Carbopol 934 (1.5%)	3:1	31.35 ± 0.74	5.65 ± 0.16	14,233.3 ± 47.1	3.50 ± 0.08	70.60 ± 0.52
G6	Carbopol 934 (2%)	1.25:1	29.30 ± 0.30	5.62 ± 0.02	13,033.3 ± 124.7	3.33 ± 0.09	72.91 ± 0.98
G7	Na-CMC (5%)	3.5:1	31.85 ± 0.29	6.22 ± 0.09	26,500.0 ± 216.0	1.13 ± 0.12	51.54 ± 1.61
G8	Sodium alginate (5%)	1.25:1	31.24 ± 0.42	6.27 ± 0.13	14,266.7 ± 205.5	3.03 ± 0.05	59.19 ± 1.43
G9	HPMC (5%)	5:1	27.35 ± 0.82	6.36 ± 0.11	12,133.3 ± 47.1	4.23 ± 0.12	55.69 ± 1.19

**Table 3:** Ex-vivo permeation parameters of GM from either control GM gel or selected GM-loaded nanoemulgel formulation.

Parameter	Optimized G6 Formulation	Control GM Gel
J_ss_ (μg/cm^2^·h^−1^)	29.20 ± 4.85	4.23 ± 1.63
K_p_ × 10^−3^ (cm/h)	18.14 ± 2.86	2.12 ± 0.99
Er	6.90	-

**Table 4:** Pharmacokinetic parameters of GM following topical application of either control GM gel or optimized GM-loaded nanoemulgel formulation.

Pharmacokinetic Parameter	Optimized GM-Loaded Nanoemulgel (G6)	Control GM-Loaded Gel
C_max_ (ng/mL)	53.75 ± 6.27	9.68 ± 2.51
T_max_ (h)	4	4
K_el_ (h^−1^)	0.086 ± 0.02	0.12 ± 0.01
t_1/2_ (h)	8.03 ± 0.32	5.91 ± 0.14
AUC_0–24h_ (ng/mL·h)	666.89 ± 36.27	61.00 ± 19.35
AUC_0–∞_ (ng/mL·h)	779.24 ± 25.19	112.63 ± 12.03
MRT (h)	12.36 ± 2.44	9.69 ± 2.03

**Table 5:** Independent and response variables for GM-loaded self-NE formulations.

Independent Variables	Type	Actual Levels	
		Low	High
A: Oil concentration (%)	Numeric	10	50
B: Tween 80 concentration in S-mix (%)	Numeric	22	88
Response variables	Goal		
R_1_: Particle size (nm)	Minimize		
R_2_: Zeta potential (mV)	Maximize		
R_3_: Dissolution efficiency (%)	Maximize		

## Data Availability

Not applicable.

## Supplementary Materials

The following supporting information can be downloaded at: https://www.mdpi.com/article/10.3390/gels9060494/s1, Table S1: Solubility of glimepiride in various nanoemulsion components; Table S2: Miscibility of various surfactants and co-surfactants in the oil phase; Table S3: Preliminary study for measuring gel strength values of different GM-loaded nanoemulgel formulations using different gel bases.
