# Supplementary material for: Optimization of Potential Nanoemulgels for Boosting Transdermal Glimepiride Delivery and Upgrading Its Anti-Diabetic Activity
Source: Gels. 2023 Jun 18;9(6):494. doi: 10.3390/gels9060494 (PMC10298085; doi:10.3390/gels9060494)
Supplement: Supplementary file 1 [file gels-09-00494-s001.zip › gels-2429007-supplementary.pdf]

gels

*Supplementary Material*

# Optimization of Potential Nanoemulgels for Boosting Transdermal Glimepiride Delivery and Upgrading Its Anti-Diabetic Activity

**Marwa H. Abdallah [1,2,]*, Amr S. Abu Lila [1,2,]*, Hanan M. El-Nahas [2] and Tarek M. Ibrahim [2]**

[1] Department of Pharmaceutics, College of Pharmacy, University of Ha'il, Hail 81442, Saudi Arabia
[2] Department of Pharmaceutics, Faculty of Pharmacy, Zagazig University, Zagazig 44519, Egypt; hananelnahas@gmail.com (H.M.E.-N.); tarekmetwally333@gmail.com (T.M.I.)
* Correspondence: mh.abdallah@uoh.edu.sa (M.H.A.); a.abulila@uoh.edu.sa (A.S.A.L.)

**Table S1.** Solubility of glimepiride in various nanoemulsion components.

| **Oils** | **Solubility (mg/g)** |
|---|---|
| Peppermint oil | 3.67 ± 0.13 |
| Bergamot oil | 2.75 ± 0.91 |
| Mandarin oil | 2.22 ± 0.19 |
| Tea tree oil | 2.11 ± 0.24 |
| Eucalyptus oil | 2.02 ± 0.17 |
| Fennel oil | 1.92 ± 0.26 |
| Lavandar oil | 1.40 ± 0.11 |
| Rosemary oil | 0.82 ± 0.10 |
| Orange oil | 0.50 ± 0.24 |
| Lemon oil | 0.43 ± 0.09 |
| **Surfactants** | **Solubility (mg/g)** |
| Tween 80 | 2.94 ± 0.09 |
| Tween 20 | 1.66 ± 0.21 |
| Span 20 | 0.92 ± 0.19 |
| Span 85 | 0.50 ± 0.13 |
| Labrafil isostearique | 0.61 ± 0.08 |
| Labrafil 1944 CS | 0.12 ± 0.03 |
| Co-surfactant | **Solubility (mg/g)** |
| Transcutol P | 2.54 ± 0.20 |
| PEG 400 | 1.15 ± 0.14 |
| PG | 0.84 ± 0.29 |
| Ethanol | 0.81 ± 0.05 |
| Lauroglycol FCC | 0.32 ± 0.10 |
| Glycerin | 0.11 ± 0.05 |

**Table S2.** Miscibility of various surfactants and co-surfactants in oil phase.

| **Gel base type** | **Gel:NE ratio** | **Gel strength (sec)** |
|---|---|---|
| Carbopol 940 (1%) | 1.5:1 | 14.01 ± 0.23 |
| | 2.5:1 | 28.13 ± 0.84 |
| Carbopol 940 (1.5%) | 1.25:1 | 11.38 ± 0.88 |
| | 1.5:1 | 26.30 ± 1.02 |
| Carbopol 940 (2%) | 0.5:1 | 17.47 ± 0.10 |
| | 1:1 | 26.25 ± 0.91 |

| | | |
|---|---|---|
| | 2:1 | 8.54 ± 1.12 |
| Carbopol 934 (1%) | 3:1 | 17.95 ± 0.41 |
| | 3.5:1 | 25.26 ± 0.86 |
| | 1:1 | 10.28 ± 0.35 |
| Carbopol 934 (1.5%) | 2:1 | 18.84 ± 1.40 |
| | 3:1 | 31.35 ± 0.74 |
| Carbopol 934 (2%) | 1:1 | 15.82 ± 1.21 |
| | 1.25:1 | 29.30 ± 0.30 |
| | 3:1 | 12.89 ± 0.91 |
| Na-CMC (5%) | 3.25:1 | 21.34 ± 0.75 |
| | 3.5:1 | 31.85 ± 0.29 |
| Sodium alginate (5%) | 1:1 | 13.39 ± 0.35 |
| | 1.25:1 | 31.24 ± 0.42 |
| | 2:1 | 10.02 ± 0.96 |
| HPMC (5%) | 3:1 | 19.29 ± 1.11 |
| | 5:1 | 27.35 ± 0.82 |

**Table S3.** Preliminary study for measuring gel strength values of different GM-loaded nanoemulgel formulations using different gel bases.

| **Oil/Surfactant** | **Number of additions (5 μL)** |
|---|---|
| Peppermint oil + (tween 80/water) | 7 additions of oil |
| Peppermint oil + (tween 20/water) | 6 additions of oil |
| Bergamot oil + (tween 80/water) | 55 additions of oil |
| Bergamot oil + (tween 20/water) | 50 additions of oil |
